# Neutrophil cell surface receptors and their intracellular signal transduction pathways^☆^

**DOI:** 10.1016/j.intimp.2013.06.034

**Published:** 2013-11

**Authors:** Krisztina Futosi, Szabina Fodor, Attila Mócsai

**Affiliations:** a Department of Physiology, Semmelweis University School of Medicine, 1094 Budapest, Hungary; b Department of Computer Science, Corvinus University of Budapest, 1093 Budapest, Hungary

**Keywords:** Abl, Abelson leukemia proto-oncogene, ADAP, adhesion and degranulation promoting adapter protein (Fyb, SLAP-130), Asc, apoptosis-associated speck-like protein containing a CARD, BCR, B-cell receptor, C3G, Crk SH3 domain-binding guanine nucleotide exchange factor (RapGEF1), CALDAG-GEFI, calcium and DAG-regulated guanine nucleotide exchange factor I, CARD, caspase activation and recruitment domain, CEACAM3, carcinoembryonic antigen-related cell adhesion molecule 3 (CD66b), CHO, Chinese hamster ovary cells, cIAP, cellular inhibitor of apoptosis, CLEC, C-type lectin, DAG, diacyl-glycerol, DAP12, DNAX activating protein 12, DISC, death-inducing signaling complex, Epac1, exchange protein activated by cyclic AMP 1, ERK, extracellular signal-regulated kinase, ERM, ezrin-radixin-moesin, ESL-1, E-selectin ligand 1, FADD, Fas-associated protein with death domain, FcR, Fc-receptor, FcRγ, Fc-receptor γ-chain, Fgr, Gardner–Rasheed feline sarcoma proto-oncogene, fMLP, formly-Met-Leu-Phe, GAP, GTPase activating protein, G-CSF, granulocyte colony-stimulating factor, GM-CSF, granulocyte/monocyte colony-stimulating factor, GPCR, G protein-coupled receptor, GPI, glycosylphosphatidylinositol anchor, GRK, GPCR kinase, Hck, hematopoietic cell kinase, ICAM-1, intercellular adhesion molecule 1, IFN, interferon, IKK, IκB kinase, IL, interleukin, IP_3_, inositol-tris-phosphate, IRAK, IL-1 receptor-associated kinase, IRF, IFN regulatory factor, ITAM, immunoreceptor tyrosine-based activation motif, IκB, inhibitor of NF-κB, JAK, Janus kinase, JNK, c-Jun N-terminal kinase, LAD, leukocyte adhesion deficiency, LFA-1, lymphocyte function-associated receptor 1 (α_L_β_2_ integrin), LTβ, lymphotoxin β, LTB_4_, leukotriene B_4_, Mac-1, macrophage antigen 1 (α_M_β_2_ integrin), MAP kinase, mitogen-activated protein kinase, MAPKAP-kinase, MAP kinase-associated protein kinase, Mcl, macrophage C-type lectin, MDA5, melanoma differentiation-associated protein 5, MDL-1, myeloid DAP12-associating lectin 1, MIP, macrophage inflammatory protein, MKK, MAP kinase kinase, MyD88, myeloid differentiation protein 88, NF-κB, nuclear factor κB, NLRP3, NOD-like receptor family, pyrin domain containing 3, NOD, nucleotide-binding oligomerization domain containing protein, OSCAR, osteoclast-associated receptor, PAF, platelet activating factor, PAK, p21-activated kinase, PI3K, phoshoinositide-3-kinase, PIP_3_, phosphatidylinositol-3-phosphate, PIR, paired immunoglobulin-like receptor, PKB, protein kinase B, PKC, protein kinase C, PLC, phospholipase C, PSGL-1, P-selectin glycoprotein ligand, Rac, Ras-related C3 botulinum toxin substrate, RANK, receptor activator of NF-κB, Rap, Ras-related protein, RIG, retinoic acid-inducible gene, RIP3, receptor-interacting serine-threonine protein kinase 3, ROS, reactive oxygen species, SAP130, Sin3A-associated protein of 130 kDa, SH2, Src-homology 2 domain, SHP-1, SH2 domain-containing protein tyrosine phosphatase 1, SLP-76, SH2 domain-containing leukocyte protein of 76 kDa, SOCS, suppressor of cytokine signaling, Src, Rous sarcoma virus proto-oncogene, STAT, signal transducer and activator of transcription, Syk, spleen tyrosine kinase, TAK, TGFβ-activated kinase 1, TCR, T-cell receptor, TGFβ, transforming growth factor β, TLR, Toll-like receptor, TNF, tumor necrosis factor, TRADD, TNFR1-associated death domain protein, TRAF, TNF receptor-associated factor, TRAIL, TNF-related apoptosis-inducing ligand, TREM, triggering receptor expressed on myeloid cells, Tyk2, tyrosine protein kinase 2, VASP, vasodilator-stimulated phosphoprotein, VCAM-1, vascular cell adhesion molecule 1, VLA-4, very late antigen 4 (α_4_β_1_ integrin), ZAP-70, ζ-chain-associated protein of 70 kDa, Neutrophils, Signaling, Receptors, Kinases, Inflammation

## Abstract

Neutrophils play a critical role in the host defense against bacterial and fungal infections, but their inappropriate activation also contributes to tissue damage during autoimmune and inflammatory diseases. Neutrophils express a large number of cell surface receptors for the recognition of pathogen invasion and the inflammatory environment. Those include G-protein-coupled chemokine and chemoattractant receptors, Fc-receptors, adhesion receptors such as selectins/selectin ligands and integrins, various cytokine receptors, as well as innate immune receptors such as Toll-like receptors and C-type lectins. The various cell surface receptors trigger very diverse signal transduction pathways including activation of heterotrimeric and monomeric G-proteins, receptor-induced and store-operated Ca^2 +^ signals, protein and lipid kinases, adapter proteins and cytoskeletal rearrangement. Here we provide an overview of the receptors involved in neutrophil activation and the intracellular signal transduction processes they trigger. This knowledge is crucial for understanding how neutrophils participate in antimicrobial host defense and inflammatory tissue damage and may also point to possible future targets of the pharmacological therapy of neutrophil-mediated autoimmune or inflammatory diseases.

## Highlights

• Neutrophils are crucial players in innate and adaptive immunity.
• Neutrophils also participate in autoimmune and inflammatory diseases.
• Various neutrophil receptors recognize pathogens and the inflammatory environment.
• The various cell surface receptors trigger diverse intracellular signaling.
• Neutrophil receptors and signaling are potential targets in inflammatory diseases.

## 1 Introduction

Neutrophils are the most abundant circulating leukocytes in the human blood. They develop in the bone marrow from the myeloid hematopoietic system and share a number of characteristic features with other myeloid cells such as monocytes/macrophages and mast cells [1,2]. Neutrophils are short-lived, terminally differentiated cells that, unless activated by a microbial or inflammatory stimulus, only survive for a short time in the bloodstream and die by a spontaneous apoptotic program, followed by removal of dead neutrophils by macrophages. Neutrophils show a condensed and multilobed nuclear morphology (likely reflecting the limited transcriptional activity of the cells) and contain a large number of intracellular granules and vesicles with no prominent staining characteristics [3]. Those features explain the alternative designation of the cells as polymorphonuclear cells or neutrophilic granulocytes.

The primary role of neutrophils is host defense against bacterial and fungal pathogens, providing the first line of defense against invading microorganisms. Neutrophils express a large number of cell surface receptors for the recognition of microbial invasion. Some of those receptors are capable of innate recognition of microbial structures while others (such as Fc-receptors) are linked to the activation of the adaptive immune response, and yet other receptors recognize the inflammatory environment.

The antimicrobial activity of neutrophils relies on the effective recognition and elimination of microbial pathogens, as well as complex intracellular signal transduction pathways linking those processes to each other. Additional signal transduction processes are not directly involved in microbial recognition and elimination but inform the cells of their environment (such as an inflammatory interstitium) or promote additional processes (such as chemotaxis) indirectly required for the elimination of pathogens. Taken together, intracellular signal transduction processes need to convey a large amount of complex information to support an efficient antimicrobial immune response.

There are several classes of receptors expressed on the surface of neutrophils, including G-protein-coupled seven-transmembrane receptors, Fc-receptors, adhesion molecules like selectins/selectin ligands and integrins, various cytokine receptors, as well as innate immune receptors including Toll-like receptors and C-type lectins (Table 1). Activation of those receptors leads to complex cellular activation and elimination processes such as phagocytosis, exocytosis of intracellular granules, production of reactive oxygen species, release of neutrophil extracellular traps, as well as additional responses like chemotactic migration or chemokine and cytokine release.

The aim of this review is to provide an overview of neutrophil cell surface receptors and their intracellular signal transduction processes. Given the very large amount of information available on that subject, only a small portion of the available data will be discussed, focusing on pathways where genetic data from primary mammalian neutrophils are available and where results may have implications in the understanding, diagnosis and therapy of autoimmune and inflammatory diseases.

## 2 Signaling by G-protein-coupled receptors

### 2.1 G-protein-coupled receptors on neutrophils

Neutrophils express a large number of G-protein-coupled receptors (GPCRs) that participate in host defense and inflammation (Table 1). Those include formyl-peptide receptors [4–6] that sense bacterial products and tissue injury (through recognition of release of mitochondrially synthesized proteins), receptors for a diverse set of “classical chemoattractants” such as leukotriene B_4_ (LTB_4_), platelet activating factor (PAF) and complement fragment C5a [6–9], as well as CXC (CXCR1, CXCR2) and, to a lesser extent, CC (CCR1, CCR2) chemokine receptors [10–13].

A common feature of the above G-protein-coupled receptors is that they strongly activate the chemotactic migration of neutrophils; therefore their agonists are conventionally termed “chemoattractants”. It should nevertheless be stated that most of those ligands (especially formyl-peptides, lipid mediators and C5a) also trigger neutrophil responses other than chemotaxis, including ROS production and exocytosis of intracellular granules and vesicles, and they are also able to augment the responses of neutrophils to subsequent stimulation by other agonists (“priming” effect).

### 2.2 GPCR signal transduction

**Fig. 1:**
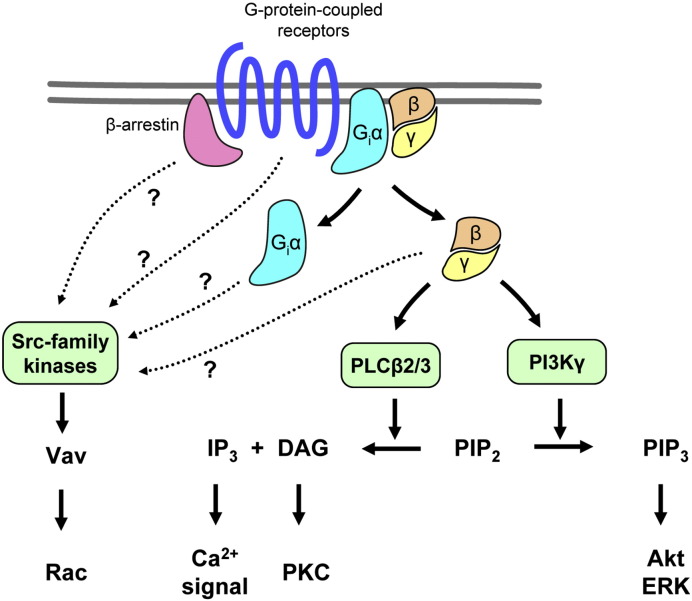
G-protein-coupled receptor signaling in neutrophils. G-protein-coupled receptors in neutrophils primarily signal through the Gβγ heterodimer, activating two parallel pathways through PLCβ2/3 and PI3Kγ. The activation of Src-family kinases likely proceeds through (an) independent and yet incompletely understood pathway(s) (question marks). See the text for further details.

All of the above GPCR agonists signal through pertussis toxin-sensitive heterotrimeric G-proteins of the G_i/o_ family. Activation of those receptors triggers the dissociation of the GPCR-specific Gα subunit from the shared Gβγ dimer and concomitant activation of various signal transduction pathways by both G-protein fragments (Fig. 1). The Gα_i_ subunit inhibits adenylyl cyclase activity and therefore reduces cytoplasmic cAMP levels. However, it is unclear whether that inhibition plays any major role in GPCR signaling in neutrophils. Instead, our current understanding is that the majority of GPCR signal transduction in neutrophils occurs through the Gβγ subunit [14–16].

One of the classical signals triggered by GPCRs in neutrophils is a prominent biphasic Ca^2 +^-signal. The first phase of this signal is likely mediated by phospholipase Cβ (PLCβ) enzymes leading to the generation of IP_3_ and concomitant release of Ca^2 +^ from intracellular stores. Indeed, the combined genetic deficiency of PLCβ2 and PLCβ3 completely abrogated fMLP-induced IP_3_ production, the increase of cytoplasmic Ca^2 +^-concentration, the activation of conventional PKC isoforms and the release of superoxide [17]. It should be mentioned that PLCβ isoforms (primarily PLCβ1) were traditionally thought to be only activated by the Gα_q_ subunit of G_q_ family heterotrimeric G-proteins. However, it was later shown that other PLCβ isoforms (particularly PLCβ2 and PLCβ3) can also be directly activated by Gβγ subunits [18–23], indicating a novel, Gα-independent PLC activation mechanism. Such a mechanism is further supported by the fact that pharmacological disruption of the Gβγ dimer inhibits GPCR-mediated chemotactic migration of neutrophils [16]. Interestingly, PLCβ2^−/−^PLCβ3^−/−^ double knockout neutrophils migrated normally towards both fMLP and MIP-1α (CCL3), indicating that PLCβ enzymes (and, likely, an IP_3_-mediated Ca^2 +^-signal) are not required for GPCR-induced neutrophil chemotaxis [17].

Another prominent pathway triggered by neutrophil GPCRs is the activation of phosphatidylinositol (PtdIns) 3-kinases (PI3-kinases or PI3K) and subsequent production of PtdIns(3,4,5)P_3_ (PIP_3_) lipid moieties (Fig. 1). Similar to PLCβ activation, PI3K-activation by neutrophil GPCRs also occurs primarily through Gβγ subunits, through the unique PI3Kγ isoform which is directly activated by Gβγ dimers [24]. Indeed, neutrophils isolated from mice deficient in the catalytic subunit of PI3Kγ showed defective PtdIns(3,4,5)P_3_ production and activation of PKB/Akt, the ribosomal S6 kinase and the ERK pathway upon stimulation by various GPCR agonists including fMLP, C5a and IL-8 [17,25,26]. PI3Kγ^−/−^ neutrophils were also defective in migrating towards fMLP, C5a, IL-8 or MIP-1α and showed defective respiratory burst upon activation by fMLP or C5a [17,25,26]. On the other hand, PI3Kγ was not required for GPCR-induced Ca^2 +^-signals [17,25,26] or fMLP-induced PKC activation [17].

The above results indicate that Gβγ subunits released upon GPCR ligation in neutrophils directly triggers two parallel receptor-proximal signal transduction events: activation of the PLCβ2/3 proteins triggers a Ca^2 +^ signal and activation of conventional PKC isoforms whereas activation of PI3Kγ leads to PIP_3_ production and PKB/Akt activation (Fig. 1). The PI3Kγ pathway (but not PLCβ2/3) is required for chemotaxis of the cells while both pathways are required for GPCR-induced superoxide release.

Prior pharmacological studies also indicated that tyrosine kinases may be involved in GPCR signaling in neutrophils [27]. Neutrophils express three members of the Src tyrosine kinase family: Hck, Fgr and Lyn. We and others found that Hck^−/−^Fgr^−/−^ double or Hck^−/−^Fgr^−/−^Lyn^−/−^ triple mutant neutrophils fail to release their intracellular granules or produce superoxide upon stimulation with fMLP [28–30]. Deficiency of Src-family kinases reduced the fMLP-induced activation of the JNK and p38 MAP kinases [29,30], as well as the activation of the Vav-Rac-PAK pathway [30] but it did not affect Ca^2 +^ signaling or Akt phosphorylation [30]. The mechanism of Src-family kinase activation by neutrophil GPCRs is at present poorly understood (see question marks in Fig. 1). A prior study indicated that Src-family kinases are activated by β-arrestins directly coupled to the chemokine receptor CXCR1 in granulocytes [31] and direct interactions between Src-family kinases and G-protein-coupled receptors or G-protein subunits have also been proposed in other cell types [32,33]. Taken together, activation of Src-family kinases by G-protein-coupled receptors in neutrophils likely occurs parallel to the PLCβ and PI3Kγ pathways, possibly mediated by the direct interaction of Src-family kinases with β-arrestins, G-protein subunits or the G-protein-coupled receptors themselves (Fig. 1).

In contrast to the role of Src-family kinases in fMLP-induced degranulation and the respiratory burst, their role in neutrophil migration is rather controversial. While Hck^−/−^Fgr^−/−^ and Hck^−/−^Fgr^−/−^Lyn^−/−^ neutrophils failed to migrate towards 2 μM fMLP in an in vitro Transwell system [30,34], the Hck^−/−^Fgr^−/−^Lyn^−/−^ cells migrated even better than wild type cells at higher doses of fMLP [34] and migration of human neutrophils toward fMLP or IL-8 in a similar Transwell system was not affected by dasatinib, a multi-specificity tyrosine kinase inhibitor, at doses where complete inhibition of Src-family kinases is expected [35]. Furthermore, Hck^−/−^Fgr^−/−^Lyn^−/−^ neutrophils migrated normally in an in vivo thioglycollate-induced peritonitis experiment [34] and the accumulation of neutrophils in that assay was not affected by the per os administration of dasatinib either (K. F. and A. M., unpublished observations). Taken together, Src-family kinases do not appear to make a major contribution to neutrophil migration.

Prior studies using pharmacological approaches and heterologous expression systems also suggested the role of the Syk tyrosine kinase in GPCR signal transduction (see references in [36]). However, our own studies using Syk^−/−^ neutrophils did not reveal any substantial defect in GPCR-induced functional or signaling responses upon the complete genetic deficiency of Syk in neutrophils or mast cells [36], whereas those cells were completely defective in signaling through β_2_-integrins or Fc-receptors [34,36]. Therefore, it is unlikely that Syk is a major component of GPCR signal transduction in neutrophils.

The ERK and p38 MAP kinases are robustly activated upon stimulation of neutrophils with G-protein-coupled receptor agonists. Prior pharmacological studies suggested a positive role for the p38 MAP-kinase pathway in GPCR signaling in neutrophils [29,37] and a recent study showed that p38 MAP-kinase promotes neutrophil migration by interfering with GRK2-mediated desensitization of formyl-peptide receptors [38]. Unfortunately, the phenotype of neutrophils lacking MAPKAP-kinase 2, the major target of p38 MAP-kinases, is rather controversial [37,39]. The functional role of the ERK pathway in neutrophil GPCR signaling is mostly unclear, in part because of a number of contradicting results in the literature [27,38,40–42].

It should also be mentioned that a few other papers have reported additional controversial studies related to the above signaling pathways. For example, one study indicated that PI3-kinases were not required for long-term chemotaxis towards fMLP [37] whereas another report suggested that PI3Kγ in neutrophils is required for something else other than GPCR-mediated gradient sensing [43]. Also, Hck and Fgr were proposed to be negative, rather than positive regulators of chemokine receptor signal transduction [44].

## 3 Fc-receptor signaling in neutrophils

### 3.1 Fc-receptor expression on neutrophils

**Fig. 2:**
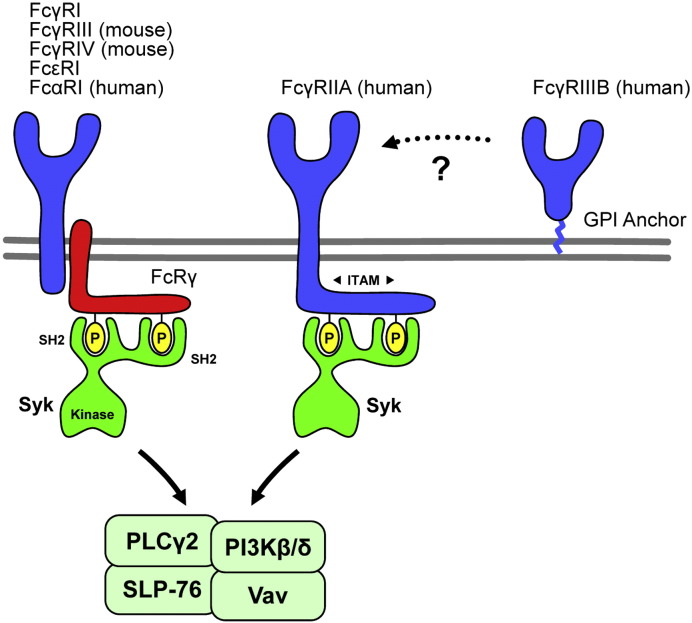
Neutrophil Fc-receptors. Low-affinity activating Fcγ-receptors signal through cytoplasmic ITAM motifs which recruit the Syk tyrosine kinase and activate further signaling. Most ITAM-coupled Fc-receptors (except FcγRIIA) are noncovalently linked to the FcRγ adapter. The human FcγRIIIB receptor has no transmembrane segment and it is linked to the membrane by a GPI anchor. See the text for further details.

Neutrophils express various Fc-receptors that are primarily involved in the recognition of Ig-opsonized pathogens but also participate in immune complex-mediated inflammatory processes (Table 1). The most important Fc-receptors in neutrophils are the low-affinity Fcγ-receptors [45]. Human neutrophils express FcγRIIA, a single-chain transmembrane receptor which carries an immunoreceptor tyrosine-based activation motif (ITAM; see below) in its cytoplasmic tail, as well as FcγRIIIB, an entirely extracellular molecule which is anchored to the plasma membrane by a GPI moiety (Fig. 2). In contrast, mouse neutrophils express FcγRIII and FcγRIV which are both multimeric receptors non-covalently associated with a transmembrane adaptor protein, the Fc-receptor γ-chain (FcRγ), which carries an ITAM motif in its intracellular tail. Since this non-covalent association is required for the stabilization of the receptor complex, the FcRγ-associated receptors are not expressed on the cell surface in the absence of FcRγ (e. g. on leukocytes of FcRγ^−/−^ animals).

Low-affinity Fcγ-receptors play important roles in immune complex-mediated activation of neutrophils. Activation of human neutrophils by immune complexes requires both FcγRIIA and FcγRIIIB (see [46] and references therein). It has been proposed that FcγRIIIB makes initial contact and tethering to immune complexes in vivo [47], followed by full activation of the cells through a synergistic ligation of both FcγRIIA and FcγRIIIB [48]. In case of mouse neutrophils, immune complex-induced cell activation is mediated by the FcRγ-associated FcγRIII and FcγRIV which function in an overlapping manner, i. e. both receptors need to be deleted or blocked to obtain complete inhibition of the responses of the cells [46].

Neutrophils also express Fc-receptors other than low-affinity Fcγ-receptors. Activated but not resting neutrophils express the high-affinity FcγRI molecule [49,50] which is of significant diagnostic value [51], but its functional relevance is poorly understood. Human (but not murine) neutrophils express FcαRI, an FcRγ-associated receptor for monomeric serum IgA [52,53]. Though this receptor is able to mediate IgA-induced inflammatory processes and tumor cell killing [54,55], the role of neutrophil Fcα-receptors in the general immune and inflammatory response is poorly understood. Under certain conditions, neutrophils likely also express Fcε-receptors [56,57] which may participate in allergic responses [57,58] or as pathogenic factors in certain infectious diseases [59], though the role of FcεRI of neutrophils has been debated by other investigators [60]. An inhibitory Fc-receptor, FcγRIIB [45], is also expressed by murine and human neutrophils and participates in negative regulation of neutrophil activation. Due to the paucity of neutrophil-specific information, we will limit the discussion below to signal transduction by low-affinity Fcγ-receptors.

### 3.2 Signal transduction by neutrophil Fc-receptors

Similar to other cell types, low-affinity activating Fcγ-receptors on neutrophils are thought to signal through the ITAM motifs present in the cytoplasmic region within the receptor complex (Fig. 2). Those motifs are short consensus sequences of YxxL/Ix_(6–12)_YxxL/I where x denotes any amino acid. The single-chain human FcγRIIA receptor contains an intrinsic ITAM whereas murine FcγRIII and FcγRIV are non-covalently linked to the ITAM-containing FcRγ adapter [45]. Crosslinking of the receptors leads to dual tyrosine phosphorylation of the ITAM sequence which then recruits the Syk tyrosine kinase to the receptor complex through binding of the two tandem SH2-domains of Syk to the two phosphorylated ITAM tyrosines [61]. This triggers the activation of Syk which will phosphorylate various tyrosine kinase substrates, therefore initiating further downstream signaling (Fig. 2). This mechanism is conceptually very similar to ITAM-mediated signal transduction by antigen receptors of B- and T-cells [61].

The mechanisms leading to ITAM phosphorylation upon Fc-receptor ligation and the kinases involved are not completely understood. Src-family kinases play a critical role in ITAM phosphorylation and activation of the Syk-related ZAP-70 kinase in T-cells [62]. However, unlike ZAP-70 which is incapable of phosphorylating the TCR-associated ITAM sequences, Syk has been shown to be able to phosphorylate ITAM sequences and it has been proposed that Syk-mediated signal transduction can be initiated even in the absence of Src-family kinase activity. Indeed, while ZAP-70-mediated receptor-proximal TCR signaling is completely blocked in the absence of the Lck tyrosine kinase, Syk activation can proceed even in the absence of Src-family kinases [63]. Also, BCR-mediated ITAM phosphorylation in B-cells is not affected by the combined deficiency of Blk, Fyn and Lyn [64] and macrophages lacking the Src-family kinases Hck, Fgr and Lyn show only delayed and modestly reduced phagocytosis of IgG-coated red blood cells whereas Syk-deficiency leads to complete loss of phagocytosis under identical conditions [65–67]. Since no studies on Fc-receptor signaling in Src-family-deficient neutrophils have yet been reported, the role of those kinases in neutrophils is at present incompletely understood. It should nevertheless be mentioned that dasatinib, a multi-kinase inhibitor with strong effects on Src-family kinases, robustly inhibits immune complex-induced activation of human neutrophils [35].

Triggering of Fcγ-receptors on neutrophils also requires the activation of a number of further signal transduction pathways. The SLP-76 adapter molecule, which was originally identified as a component of T-cell receptor signal transduction, was shown to be required for FcγR-mediated Ca^2 +^-flux and superoxide production [68]. The PLCγ2 phospholipase was also essential for immune complex-mediated activation of neutrophils, likely downstream of Src-family kinases and Syk [69]. Fcγ-receptor-mediated neutrophil activation required members of the Vav guanine nucleotide exchange factor and Rac small GTPase families with predominant roles for Vav3 and Rac2 [70,71]. Immune complex-induced neutrophil activation also required the PI3-kinase isoforms PI3Kβ and PI3Kδ with a predominant role for PI3Kβ [72].

Given the association of Fcα-receptors to the FcRγ adapter, it is expected that those receptors also signal through an ITAM-dependent mechanism [52,53].

## 4 Signaling by selectins/selectin ligands and integrins

### 4.1 Neutrophil adhesion receptors

The two major groups of neutrophil adhesion receptors are selectins/selectin ligands and integrins (Table 1).

Selectins are single-chain transmembrane glycoproteins that recognize carbohydrate moieties and mediate transient interactions between leukocytes and the vessel wall [73]. P-selectin is expressed on platelets and endothelial cells with increased endothelial expression in an inflammatory environment. E-selectin is expressed on endothelial cells but only under inflammatory conditions. L-selectin is expressed on leukocytes. Selectins interact with a large number of carbohydrate-containing cell surface molecules including the best characterized P-selectin glycoprotein ligand 1 (PSGL-1), a mucin-like protein expressed on the leukocyte surface, which is the counterreceptor of P- and E-selectins on endothelial cells [74]. However, endothelial E-selectins also bind to other selectin ligands on the leukocyte surface, including CD44 and ESL-1 [75], as well as (in case of human leukocytes) various glycolipid ligands [76]. Selectins and selectin ligands are required for the rolling phase of the leukocyte adhesion and transmigration cascade (see below). More detailed description of selectins/selectin ligands and the role of carbohydrate-recognizing receptors in immune cell trafficking can be found in excellent recent reviews [73,74].

Integrins are heterodimeric transmembrane glycoproteins present on virtually all mammalian cells. The most important integrins expressed on leukocytes belong to the β_2_ integrin family, formed by the β_2_ (CD18) integrin chain and a unique α chain [77]. LFA-1 (α_L_β_2_; CD11a/CD18) is expressed on all circulating leukocytes while Mac-1 (α_M_β_2_; CD11b/CD18) is primarily expressed on myeloid cells such as neutrophils, monocytes and macrophages. LFA-1 and Mac-1 bind to endothelial ICAM-1 and are involved in different phases of neutrophil adhesion and transendothelial migration (see below). Leukocytes also express the VLA-4 (α_4_β_1_) integrin which binds to endothelial VCAM-1. The role of this interaction is well known in the case of lymphocytes but less well characterized in the case of neutrophils (see [78] and references therein). A more detailed description of the functions of β_2_-integrins in neutrophils can be found in a recent review [77].

### 4.2 The neutrophil adhesion and transendothelial migration cascade

**Fig. 3:**
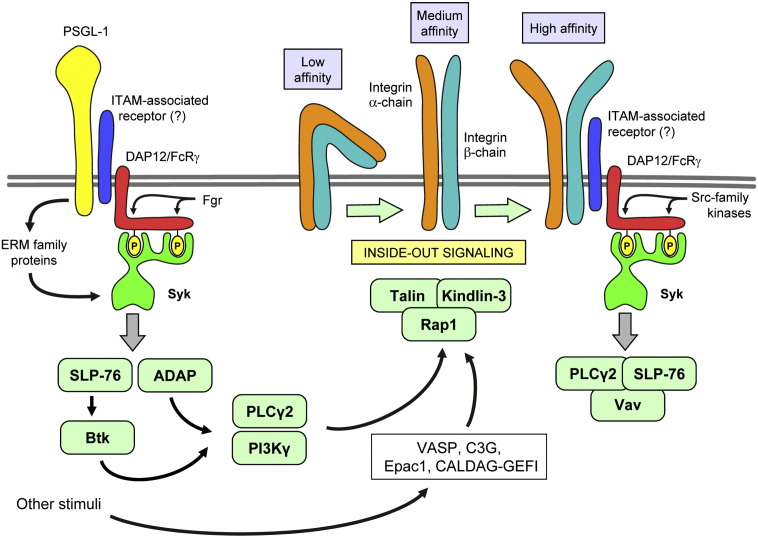
Signal trandsuction by selectin ligands and integrins. PSGL-1 and β_2_-integrins signal through an ITAM-based mechanism, involving the DAP12 and FcRγ adapter proteins and the Syk tyrosine kinase. Additional signaling proteins are involved in the regulation of the integrin binding affinity (inside-out signaling). See the text for further details.

While the majority of neutrophils circulate in the bloodstream under resting conditions, microbial invasion or other inflammatory stimuli trigger the extravasation of neutrophils to the inflamed interstitium. This process is mediated by a multistep cascade of neutrophil adhesion to, and transmigration through, the vessel wall [79]. Under resting conditions, neutrophils make temporary, reversible contacts with the endothelium which leads to a characteristic rolling phenomenon with an average speed of approx. 40 μm/s (steady-state rolling). This steady-state rolling is primarily mediated by the interaction of endothelial P-selectins with their neutrophil glycoprotein counterreceptors, primarily PSGL-1. The rolling velocity of neutrophils is dramatically reduced to approx. 5 μm/s in an inflammatory environment (slow rolling). This deceleration is due to the expression of E-selectins on the inflamed endothelium which provides increased number of binding sites for PSGL-1 and also triggers an intermediate-affinity conformational state of the β_2_-integrin LFA-1 on neutrophils (Fig. 3). This leads to further neutrophil–endothelial cell interactions through the binding of LFA-1 to its endothelial counterreceptor ICAM-1 during the slow rolling phase [80–82]. Therefore, unlike steady-state rolling, slow rolling is mediated by both selectins and integrins [79]. The inflamed endothelium also expresses a number of other cell surface molecules (such as membrane-bound chemokines and cytokines) that trigger further neutrophil activation. An important result of that neutrophil activation is the development of a high-affinity conformation of the neutrophil integrin LFA-1 (and, possibly, other integrins such as VLA-4 and Mac-1), leading to increased binding to endothelial integrin ligands (such as the LFA-1 ligand ICAM-1 and, possibly, the VLA-4 ligand VCAM-1). This results in full arrest of the neutrophils at the endothelial surface. Arrested neutrophils begin to spread over the endothelium which results in adhesion strengthening and firm adhesion [79]. Before leaving the vessel lumen, neutrophils crawl on the endothelium, primarily using cell surface Mac-1 integrins binding to endothelial ICAM-1. After finding the place for transmigration, neutrophils migrate to the interstitium through transcellular or paracellular routes and begin chemotaxing towards the site of infection/inflammation within the perivascular and interstitial space. Those processes also require β_2_-integrins and other, not fully characterized adhesion receptors [79].

The role of β_2_-integrins and carbohydrate-binding receptors (primarily selectins) is well represented in the various forms of leukocyte adhesion deficiency (LAD), a severe inherited leukocyte adhesion and migration defect resulting in severe bacterial infections in human patients [83]. While LAD Type 1 is caused by the deficiency of the β_2_ integrin chain CD18, LAD Type 2 is caused by a defect in cellular fucose metabolism, leading to defective selectin-mediated cell–cell interactions [83]. LAD Type 3 is due to defective inside-out signaling of various integrins including leukocyte β_2_-integrins (see below).

### 4.3 Signal transduction by neutrophil selectins and selectin ligands

Though the interaction between selectins and selectin ligands is very short and primarily determined by the molecular interactions between the extracellular portions of the molecules, selectin-mediated interactions also trigger intracellular signal transduction processes. The principal example of this is the increased LFA-1-mediated adhesion of leukocytes following PSGL-1 binding to E-selectin [80–82]. PSGL-1 induces an intermediate affinity state of LFA-1 by mechanisms resembling immunoreceptor (e. g. Fc-receptor) signal transduction (Fig. 3) whereby Syk is activated through ITAM-bearing molecules such as the DAP12 and FcRγ adapters [84,85] and ERM family proteins which also contain ITAM-like motifs [86]. The activation of Syk also requires the Src-family kinase Fgr which is likely responsible for phosphorylation of the ITAM tyrosines of DAP12 and FcRγ [85]. Accordingly, Hck^−/−^Fgr^−/−^ and Hck^−/−^Fgr^−/−^Lyn^−/−^ neutrophils show reduced binding to E-selectin-expressing CHO cells [87]. PSGL-1 signaling then activates the closely coupled SLP-76 and ADAP adaptors which then activate the Btk tyrosine kinase [88]. The signaling then diverges into a PLCγ2- and PI3Kγ-mediated pathway (Fig. 3), both of which are required for PSGL-1-mediated LFA-1 activation and inflammation-induced slow rolling of neutrophils [89]. The final steps of PSGL-1-mediated LFA-1 activation involve the Rap1 small GTPase by CALDAG-GEFI [90], as well as talin-1 [91]. The steps of LFA-1 regulation by selectin-mediated signal transduction in neutrophils have been discussed in detail in excellent recent reviews [92,93].

### 4.4 Signal transduction by neutrophil integrins

Integrin signaling can be divided into signals triggered by integrin ligation (outside-in signaling) and the regulation of integrin ligand binding (e. g. affinity) by intracellular signals (inside-out signaling) [94].

Integrin outside-in signaling can be triggered by placing neutrophils on integrin ligand-coated surfaces (such as ICAM-1, fibrinogen or whole serum) in the presence of a proinflammatory stimulus (such as TNF-α or chemoattractants) [95]. This triggers cell spreading, respiratory burst and degranulation responses which are dependent on cell surface β_2_-integrins [34,96]. β_2_ integrin ligation leads to activation of Src-family kinases and the combined genetic deficiency of Hck and Fgr or Hck, Fgr and Lyn blocks β_2_ integrin-mediated functional and signaling responses of neutrophils [28,34,97,98] without affecting inside-out activation [99]. β_2_ integrin-mediated neutrophil activation also requires the Syk tyrosine kinase [34,61]. Interestingly, integrin-mediated Syk activation is mediated by two ITAM-bearing adapter proteins, DAP12 and FcRγ, in a classical phospho-ITAM-mediated manner [98] (Fig. 3). Further downstream signaling requires the SLP-76 adapter protein [68], the PLCγ2 phospholipase [69,100] and members of the Vav gunanine nucleotide exchange factor family [100,101]. These results indicate that outside-in signaling by neutrophil integrins triggers a signal transduction pathway similar to that of classical immunoreceptors (such as B- and T-cell-receptors and Fc-receptors) (Fig. 3) [61,102].

Further downstream steps of outside-in signaling by β_2_ integrins in neutrophils are less understood. Though pharmacological studies suggested a role for the Abl tyrosine kinase in that process [103], no genetic studies have yet confirmed that conclusion. The mammalian actin-bundling protein mAbp1 was shown to be activated by Syk and to mediate some of its effects in neutrophils [104]. Though p190RhoGAP was proposed to play a major role in β_2_ integrin signal transduction [105], later genetic studies using p190RhoGAP^−/−^ neutrophils failed to confirm that conclusion [106]. Neutrophils also express α_4_ integrins such as VLA-4 which also signal through Src-family kinases [78].

Significantly less is known about integrin inside-out signaling in neutrophils, likely because of the technical difficulties involved. The above-described E-selectin-mediated LFA-1 activation pathway is likely specific for E-selectin. Though Rap1 is generally believed to regulate inside-out integrin activation in various hematopoietic lineages [107,108], this has not yet been directly confirmed in neutrophils, likely in part because of the embryonic lethality of Rap1^−/−^ mice [109]. Rap1 is nevertheless activated by a number of stimuli including G-protein-coupled receptors or E-selectin ligands and it has been proposed that this is mediated by VASP, the Rap1 guanine nucleotide exchange factor C3G and Epac1 in neutrophils [110,111] (Fig. 3). CALDAG-GEFI was also shown to activate Rap1 and regulate integrin inside-out activation [112]. In addition, talin-1 and kindlin-3 are both necessary for the induction of the high-affinity LFA-1 conformation required for neutrophil arrest at the endothelium [91,113].

It should also be mentioned that the recently identified LAD Type 3 variant in humans causes defective integrin inside-out activation in neutrophils and other cell types [114–117]. After initially suspecting CALDAG-GEFI to carry the responsible mutation [112,118], it was later shown that the actual defect lies in the kindlin-3 molecule [119–121], providing additional evidence for kindlin-3 in inside-out signaling of neutrophil β_2_ integrins.

It should also be mentioned that the role of the above molecules in integrin-mediated neutrophil activation may not necessarily translate to their role in integrin-mediated neutrophil migration as indicated by mostly normal migration of neutrophils lacking Src-family kinases, Syk, ITAM-bearing adapters (DAP12 and/or FcRγ), PLCγ2 or Vav-family exchange factors [34,36,69,98,101]. Therefore, integrins may use different signal transduction pathways to trigger adherent activation and migration of neutrophils.

## 5 Cytokine receptor signal transduction

Neutrophils express a number of cytokine receptors including conventional cytokine receptors, members of the IL-1-receptor/Toll-like receptor family, and TNF-receptor family members (Table 1). Those receptors are involved in intercellular communication regulating various neutrophil functions.

### 5.1 Type I and type II cytokine receptors

Conventional cytokine receptors are grouped into type I and type II cytokine receptors (Table 1). Those are multimeric (mostly dimeric) molecules with several phosphorylatable tyrosine residues in their intracellular sequences.

Type I cytokines consist of 4 α-helices and bind to type I cytokine receptors which have a conserved extracellular WSXWS motif. The most important type I cytokine receptors expressed by neutrophils are IL-4, IL-6, IL-12 and IL-15 receptors, as well as G-CSF and GM-CSF receptors. Type I cytokine receptors are either homodimeric (e. g. G-CSF-receptor) or are heterodimers (or heteromultimers) of ligand-specific chains and common receptor chains shared with other receptors (see [122] for further details).

Type II cytokines consist of 6 α-helices and bind to type II cytokine receptors which do not contain the WSXWS motif. Important type II cytokine receptors on neutrophils are receptors for IFNα, IFNβ, IFNγ and the inhibitory IL-10 cytokine. Sharing of receptor chains between type II cytokine receptors is less common.

Type I and type II cytokine receptors are involved in a number of neutrophil functions. G-CSF and GM-CSF direct the differentiation, survival and activation of neutrophils [123]. Additional cytokines such as IL-4 [124,125], IL-6 [126–128] and IL-15 [129,130] are also involved in activation of neutrophils and the coordination of the inflammatory response. Of type II cytokines, IFNα/β (type I interferons) delay apoptosis of neutrophils [131] whereas IFNγ (type II interferon) enhances the respiratory burst, triggers gene expression changes and delays apoptosis of neutrophils [132]. IL-10, another member of the type II cytokine family, exerts an inhibitory effect on various functional responses of neutrophils, including chemokine and cytokine production [133].

**Fig. 4:**
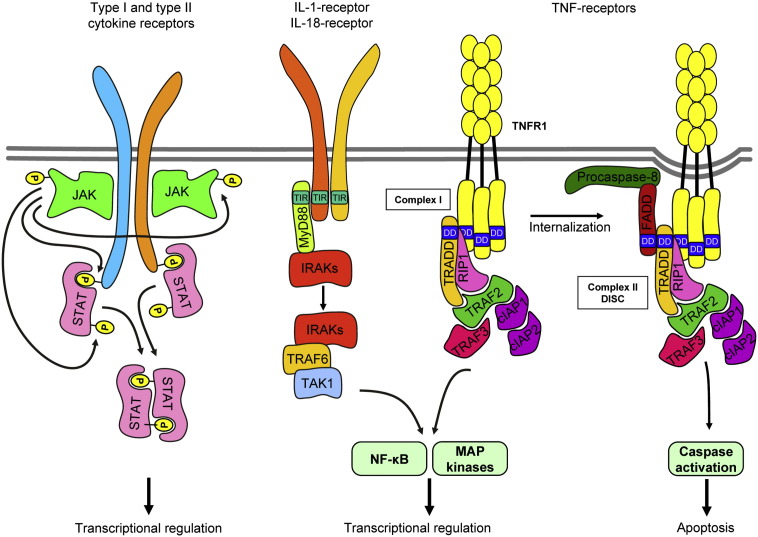
Signal transduction of cytokine receptors. Type I and type II cytokine receptors signal via activation of the JAK-STAT pathway. IL-1 and IL-18 receptors activate IRAK family proteins through MyD88. TNF-family receptors trigger two different signal transduction pathways through recruiting two different complexes of intracellular adapters (Complex I and Complex II). DD, death domain. See the text for further details.

Type I and type II cytokine receptors trigger the activation of the JAK-STAT pathway [134–136] (Fig. 4). Members of the JAK kinase family are constitutively associated with the receptor and become activated upon receptor ligation. Activated JAKs lead to phosphorylation of other JAK molecules within the receptor complex and also phosphorylate intracellular tyrosine residues on the receptor chain. This recruits STAT transcription factors from the cytoplasm which also become phosphorylated by the receptor-associated JAK-family kinases. Phosphorylated STATs are then released from the receptor, dimerize and shuttle to the nucleus where they bind to cognate DNA sequences and regulate gene transcription (Fig. 4).

There is a wide diversity of JAK and STAT proteins utilized by the different cytokine receptors [135,136]. IL-4 and IL-15 receptors (which share a common γ-chain) use both Jak1 and Jak3 and activate Stat5 or Stat6. The β-chain-containing GM-CSF and the homodimeric G-CSF receptors utilize Jak2 and Stat5 or Stat3. The gp130-containing IL-6 and IL-12 receptors use various JAK proteins (most importantly Jak1 and Tyk2) and activate Stat3 or Stat4. The type II cytokine receptors for IFNs and IL-10 primarily utilize Jak1 with some accessory role for Jak2 and Tyk2, and activate Stat1, Stat2 or Stat3. An additional level of complexity may be caused by the expression of different JAK and STAT isoforms in different cellular lineages. It is at present unclear how the specificity of the receptor is carried over through different JAK and STAT family members and how a limited number of JAK and STAT components are able to trigger additional specific signals.

Type I and type II cytokine receptors also activate a number of additional signal transduction processes in neutrophils. Those include activation of Src-family kinases [137–140], the PI3-kinase-Akt pathway [138,140–142], the ERK and p38 MAP kinases [143,144], and the inhibitory SOCS molecules [145–147].

### 5.2 IL-1 receptor family

The IL-1 isoforms IL-1α and IL-1β are among the most potent cytokines and are important mediators of the inflammatory response [148]. While IL-1β is synthesized as an inactive precursor (pro-IL-1β) and is processed to its final form by an intracellular protease complex called the inflammasome, no such processing is required for release of IL-1α. Despite a major role in the overall inflammation response, IL-1 isoforms do not trigger a robust neutrophil activation and their main effect on neutrophils is to prolong the survival of the cells [149]. IL-18 is a structurally related proinflammatory cytokine which is also processed by inflammasome-mediated proteolytic cleavage. IL-18 triggers various responses of neutrophils including chemokine and cytokine release, enhanced activation of the respiratory burst and inhibition of neutrophil apoptosis [150,151], in part as an autocrine regulator of the cells [152].

Receptors for IL-1 isoforms (IL-1RI) and IL-18 (IL-18R) are members of the IL-1-receptor/Toll-like receptor (IL-1R/TLR) superfamily with Ig-like extracellular domains [153]. Both IL-1 ans IL-18 receptors consist of a principal chain and an accessory protein (IL-1RAcP and IL-18RAcP, respectively) [153]. There are two IL-1 receptors: IL-1RI which mediates the biological effects of IL-1 isoforms and IL-1RII which is a truncated receptor lacking intracellular signaling domains and works primarily as a decoy receptor [149,154]. IL-1RI binds IL-1α, IL-1β and IL-1R antagonist (IL-1Ra). Neutrophils express the IL-1 receptors [155], and the expression of these receptors is increased in septic patients [156]. Though neutrophils predominantly express the non-functional decoy receptor IL-1RII [149,157], the cells also express IL-1RI and respond to IL-1 stimulation (though not as strongly as other immune cells).

Ligand binding recruits the MyD88 adaptor protein to the TIR domain within the cytoplasmic region of IL-1RI and IL-18R, resulting in recruitment, activation and autophosphorylation of IRAK-family kinases [153] (Fig. 4). IRAKs are then released from the receptor-MyD88 complex and couple to the E3 ubiquitin ligase TRAF6 which auto-ubiquitinates itself and binds and activates TAK1. TAK1 then activates the IKK complex to release NF-κB from IκBα-mediated inhibition, and also triggers MKK enzymes resulting in activation of ERK, JNK and p38 MAP kinase pathways (Fig. 4). Accordingly, activation of the NF-κB and the ERK, JNK and p38 MAP kinase cascades can be observed in neutrophils activated by IL-18 [152,158].

### 5.3 TNF receptor family

The TNF-receptor superfamily consists of various receptors with diverse biological functions, and is divided into receptors carrying an intracellular death domain (such as TNFR-1, Fas, TRAIL-R2) and those having no death domains (such as TRAIL-R3, LTβR or RANK). TNF-α is a major cytokine triggering neutrophil activation [95,159–161] and priming of responses to additional stimuli [162,163]. Neutrophils also express the TNF receptor-related Fas [164,165], TRAIL receptors (TRAIL-R2 and TRAIL-R3) [166,167], RANK [168], and LTβ receptor [169]. Neutrophils express both the 55 kDa TNFR1 and the 75 kDa TNFR2.

TNF-receptors trigger intracellular signaling by recruiting adapter proteins to the receptor complex [170,171]. In general, while both TNFR1 and TNFR2 trigger pro-inflammatory (anti-apoptotic) signals, only TNFR1 triggers pro-apoptotic responses. The pro-inflammatory signal is mediated by the so-called complex 1, generated by the direct association of TNFR1 with TRADD and RIP1, leading to the secondary recruitment of TRAF2, TRAF3, cIAP1 and cIAP2 and eventual activation of the JNK and NF-κB pathways (Fig. 4). The same pro-inflammatory (JNK and NF-κB) pathways are also triggered by TNFR2, but in that case, TRAF2, TRAF3, cIAP1 and cIAP2 are directly recruited to the receptor itself. TNFR1 (but not TNFR2) is also able to transmit a pro-apoptotic signal by recruiting another complex (complex 2, also known as death-induced signaling complex or DISC) following conformation and ubiquitination changes and internalization of the receptor complex (Fig. 4). In that signaling pathway, RIP3, FADD, and procaspase-8 are recruited to the receptor leading to caspase activation and apoptosis [171]. This general scheme may be modified in neutrophils with the additional role of PKCδ, PI3K, p38 MAP kinase and caspase-3 activation [172,173], as well as by SHP-1-mediated disruption of anti-apoptotic signaling of G-CSF and GM-CSF [174]. In addition, TNFR1 and TNFR2 cooperate during TNF-induced respiratory burst of neutrophils [175].

### 5.4 Additional cytokines: TGFβ and IL-17

Neutrophils also respond to additional cytokines, whose function will only briefly described here. Though neutrophils express receptors for TGFβ, its importance in regulating neutrophil function is poorly understood, except for a proposed effect on functional polarization of tumor-associated neutrophils towards a pro-tumorigenic phenotype [176]. While neutrophils are among the major components of antimicrobial and inflammatory responses triggered by IL-17 family members (primarily IL-17A and IL-17F), neutrophils do not express IL-17 receptors and do not respond to IL-17 directly [177]. Instead, IL-17 triggers the release of various other cytokines (such as TNF-α, CXC chemokines and G/GM-CSF) which affect neutrophil function in an indirect manner. Further details on TGFβ-receptor signal transduction [178,179] and IL-17-mediated inflammatory responses and IL-17 receptor signaling [180–184] can be found in excellent recent reviews.

## 6 Signaling by innate immune receptors

Neutrophils express a number of innate immune receptors (so-called pattern recognition receptors) involved in the direct recognition of pathogens and tissue damage. Those include Toll-like receptors, C-type lectins, Nod-like receptors, and RIG-like receptors (Table 1). An additional group recognizing bacterial- and mitochondrial-derived formyl-peptides has been discussed in the section on GPCR signaling above.

### 6.1 Toll-like receptors in neutrophils

Toll-like receptors (TLRs) are the best known innate immune receptors present on the cell surface or in intracellular endocytic compartments [185,186]. Neutrophils express all tested TLRs except TLR3 [187–190] (the expression of TLR7 is debated [188]). Neutrophil TLRs recognize various microbial structures such as bacterial lipopolysaccharide (TLR4) or peptidoglycans (TLR2), leading to increased cytokine and chemokine production, priming and delayed apoptosis of the cells [189–192].

**Fig. 5:**
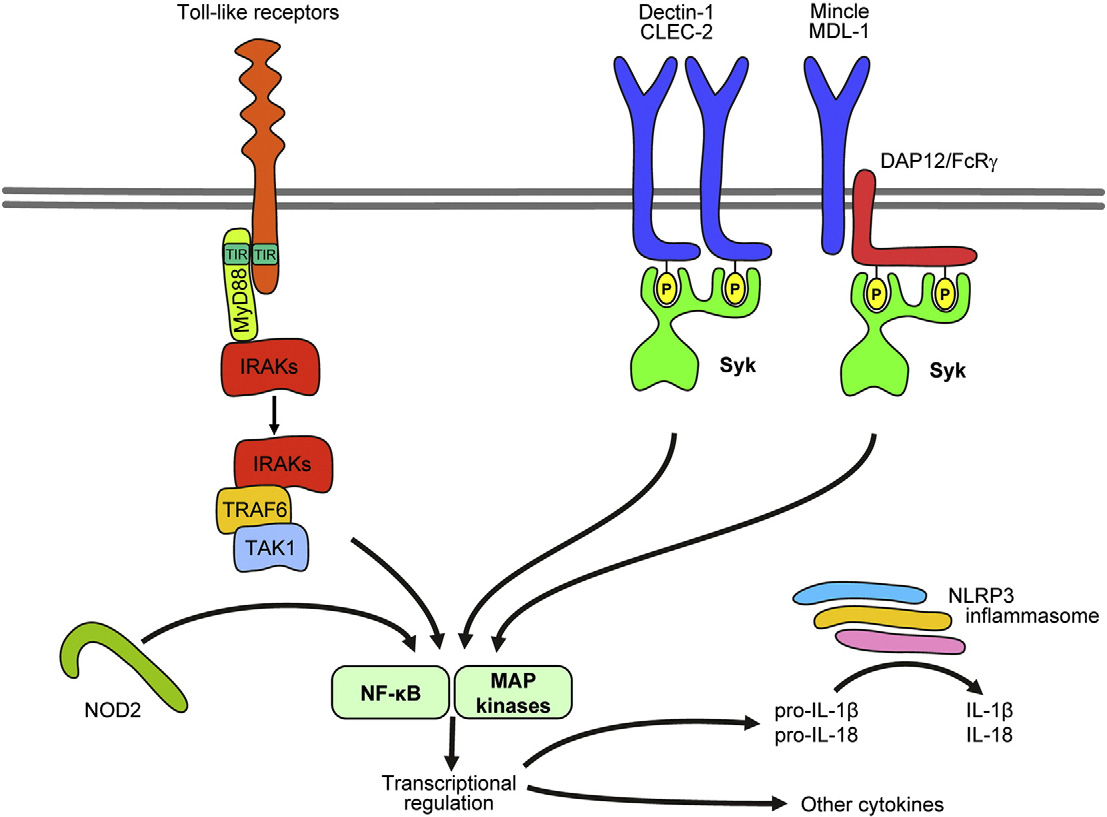
Signaling by innate immune receptors. Toll-like receptors activate IRAK family proteins through MyD88. C-type lectins signal through an ITAM-like mechanism activating Syk. NOD2 is an intracellular sensor activating the NF-κB pathway. The NLRP3 inflammasome processes pro-IL-1β and pro-IL-18 to their active form. See the text for further details.

Toll-like receptors belong to the IL-1R/TLR family with leucine-rich repeats in their extracellular domains [153]. The principal TLR signal transduction pathway is mediated by recruitment of the MyD88 adapter. MyD88 recruits IRAK family kinases (primarily IRAK4), leading to IRAK phosphorylation and further recruitment of TRAF6 and TAK1 (Fig. 5). TAK1 will then trigger activation of the NF-κB pathway through IKK, as well as the p38 and JNK pathways through MKK proteins [153,193], resulting in transcriptional regulation of cytokine production and other proinflammatory processes.

Most of the above information has been obtained from cell types other than neutrophils. The role of IRAK4 in TLR signaling in neutrophils has been confirmed by defective signaling of most TLR family members (except TLR9) in neutrophils from IRAK4-deficient patients [194,195]. TLR2-dependent IL-10 production by neutrophils was also defective in MyD88^−/−^ mouse neutrophils [196]. Besides the above components, additional players such as peroxynitrite, PI3-kinases or various MAP-kinases have also been proposed to transmit TLR signals in neutrophils [197–200].

### 6.2 C-type lectins

Neutrophils express innate immune receptors belonging to the C-type lectins, such as Dectin-1 (CLEC7A) [201,202], Mincle (CLEC4E) [203], MDL-1 (CLEC5A) [204], Mcl (CLEC4D) [205] and CLEC2 [206] (Table 1). Dectin-1 is the principal receptor for fungal β-glucans [207] and was proposed to participate in fungal recognition by neutrophils [202]. Mincle is a multifunctional receptor recognizing *Malassezia* fungi [208], mycobacterial structures [203,209] and cytoplasmic danger signals (such as SAP130) [210]. MDL-1 is likely involved in viral recognition [211] and CLEC2 is a receptor for the guidance molecule podoplanin [212,213] with little information on their role in neutrophils. The ligand and functional role of Mcl are at present mainly unknown.

Most C-type lectins signal through an ITAM-based mechanism similar to that of Fc-receptors (Fig. 5). Mincle and MDL-1 are associated with ITAM-bearing transmembrane adapters (FcRγ and DAP12, respectively) [210,214]. In contrast, Dectin-1 and CLEC-2 contain so-called hemITAMs (half of an ITAM) within the principal receptor chain which likely act similar to full ITAMs following receptor dimerization [215]. Receptor ligation leads to phosphorylation of the ITAM/hemITAM tyrosine residues, leading to recruitment and activation of Syk [61]. Mcl (which does not couple to known ITAM/hemITAM motifs) also activates Syk by a yet unknown mechanism [205]. Syk activation triggers tyrosine phosphorylation of downstream molecules including Vav-family proteins [216]. Based on studies on other Syk-coupled receptors and other cell types, it is expected that SLP-76, PLCγ2, the CARD9 adapter, NF-κB-mediated gene transcription and the NLRP3 inflammasome are also involved in signaling by C-type lectins [61,217,218].

### 6.3 NOD-like receptors

NOD-like receptors are cytoplasmic sensors of pathogens and danger signals which lead to transcriptional changes or activate cytokine-processing caspases.

NOD1 and NOD2 are sensitive to bacterial structures such as proteoglycan degradation products. Their ligation leads to ubiquitination of RICK and subsequent activation of TAK1, NF-κB and MAP-kinase pathways, triggering inflammatory cytokine production [219]. Neutrophils express NOD2 but not NOD1, and the administration of NOD2-specific (but not NOD1-specific) proteoglycan components trigger IL-8 release and cellular activation [220]. No further details of NOD2 signaling in neutrophils are available at the moment.

The NOD-like receptor NLRP3 is sensitive to bacterial products, as well as various forms of cellular damage such as ATP, uric acid or depletion of intracellular K^+^ [219]. Unlike NOD1/2, NLRP3 does not affect gene transcription but triggers the so-called NLRP3 inflammasome (consisting of NLRP3, Asc and caspase-1), leading to processing of pro-IL-1β and pro-IL-18 to their mature form by caspase-1-mediated proteolytic cleavage (Fig. 5) [221,222]. Neutrophils express all components of the NLRP3 inflammasome and genetic deficiency of its components blocks IL-1β production of neutrophils by danger signals [223].

### 6.4 RIG-like receptors

Though neutrophils were originally thought to fight exclusively against extracellular microbes, they also appear to be involved in host defense against viral pathogens (see e.g. [224]). Intracellular viruses are in part recognized by RIG-I-like receptors, a family of RNA helicases that function as cytoplasmic sensors of double-stranded RNA [225]. Upon ligation, they associate with the IPS-1 adaptor and activate interferon regulatory factors (IRF3 and IRF7) and NF-κB, triggering type I interferon production and expression of other antiviral genes [225]. Neutrophils express both RIG-I and the related MDA5 receptor [187,188], and are able to release cytokines and change gene expression when activated by poly(I:C), a synthetic mimetic of viral double-stranded RNA [187]. Poly(I:C)-induced responses of neutrophils require, among others, MAP-kinases, NF-κB and IRF3 [187].

## 7 Other receptors in neutrophils

Neutrophils also express a number of additional receptors that cannot be grouped into the above categories. Those include DAP12- and FcRγ-associated receptors such as TREM-1 [226,227] and OSCAR [228]; the *Neisseria* recognition receptor CEACAM3 [229,230]; as well as scavenger receptors, complement receptors and various intracellular lipid-sensing receptors. The signaling pathways of those receptors have been omitted from this review because of uncertainties related to their function and/or signaling in neutrophils.

Most of the above information relates to effects of activating receptors on neutrophils. However, neutrophils also express a number of inhibitory receptors which inhibit or terminate their responses. Those include the immunoreceptor tyrosine-based inhibitory motif (ITIM) containing FcγRIIB and PIR-B which likely signal through the SHP-1 tyrosine phosphatase [44,45,231], as well as the inhibitory IL-10 receptor [133]. Due to space limitation, details of inhibitory signaling in neutrophils have been omitted from this review.

## 8 Neutrophil receptors and signaling as therapeutic targets

Neutrophils participate in the development of various autoimmune and inflammatory diseases, including rheumatoid arthritis, systemic lupus erythematosus, blistering skin diseases, autoimmune vasculitides, anaphylactic reactions, as well as metabolic-vascular diseases such as atherosclerosis, thrombosis, ischemia–reperfusion injury, or even insulin resistance [232–237]. Though it is technically challenging to directly link neutrophil receptors or their signaling molecules to specific diseases, there are at least two studies indicating that neutrophil-specific expression of Fc-receptors, C5a-receptors and LFA-1 [238], as well as the Syk tyrosine kinase [239] are required for autoantibody-mediated arthritis. There are also very strong correlation between neutrophil functions and autoantibody-induced disease development in mice lacking the PI3-kinase isoforms PI3Kβ and PI3Kδ [72] or the PLCγ2 protein [69]. Genetic deficiency of Syk [239,240], as well as a novel Syk inhibitor [241] protected mice from autoantibody-induced arthritis but also prevented neutrophil activation in various assay systems [34,98,241,242]. Fostamatinib, an orally available pro-drug of that inhibitor has recently produced very promising effects in a Phase II clinical trial in human rheumatoid arthritis [243] and it is reasonable to assume that at least some of those are due to targeting Syk within the neutrophil compartment. Dasatinib, a combined Abl/Src tyrosine kinase inhibitor used for the treatment of chronic myelogenous leukemia also shows robust inhibitory effects on certain neutrophil functions [35] and may prove to be a suitable starting point of development of novel tyrosine kinase inhibitor anti-inflammatory molecules. Those and other studies suggest that neutrophil receptors and their signal transduction processes may prove to be suitable targets of the future pharmacological therapy of diseases characterized by excessive neutrophil activation.

## 9 Concluding remarks and future directions

Neutrophils are crucial players of innate immunity and inflammation and they also participate in the effector phase of adaptive immunity. Their function is mediated by a number of cell surface receptors which trigger complex intracellular signal transduction pathways that we are only beginning to understand. However, given the major role of neutrophils in various human diseases, understanding signal transduction in neutrophils is of major biomedical importance. Novel transgenic approaches allowing the lineage-specific analysis of signal transduction processes in live animals (such as a recent study showing the protective effect of neutrophil-specific deletion of Syk in the development of autoantibody-induced arthritis [239]) will provide major advances in the field, and may later be utilized for therapeutic purposes in diseases with a neutrophil-mediated pathogenetic component.

## Figures and Tables

**Table 1:** The most important neutrophil receptors. See the text for further details.

G-protein-coupled receptors	Fc-receptors	Adhesion receptors	Cytokine receptors	Innate immune receptors
*Formyl-peptide receptors* • FPR1 (FPR) • FPR2 (FPRL1) • FPR3 (FPRL2) *Classical chemoattractant receptors* • BLT1 (LTB_4_-rec.) • BLT2 (LTB_4_-rec.) • PAFR • C5aR *Chemokine receptors* • CXCR1 (human) • CXCR2 • CCR1 • CCR2	*Fcγ-receptors* • FcγRI • FcγRIIA (human) • FcγRIIB (inhibitory) • FcγRIII (mouse) • FcγRIIIB (human) • FcγRIV (mouse) *Fcα-receptors* • FcαRI (human) *Fcε-receptors* • FcεRI • FcεRII	*Selectins and selectin ligands* • L-selectin • PSGL-1 *Integrins* • LFA-1 (α_L_β_2_) • Mac-1 (α_M_β_2_) • VLA-4 (α_4_β_1_)	*Type I cytokine receptors* • IL-4R • IL-6R • IL-12R • IL-15R • G-CSFR • GM-CSFR *Type II cytokine receptors* • IFNAR (IFNα/β-rec.) • IFNGR • IL-10R *IL-1R family* • IL-1RI • IL1RII (decoy) • IL-18R *TNFR family* • TNFR1 (p55) • TNFR2 (p75) • Fas • LTβR • RANK • TRAIL-R2 • TRAIL-R3	*Toll-like receptors* • TLR1 • TLR2 • TLR4 • TLR5 • TLR6 • TLR7 (?) • TLR8 • TLR9 *C-type lectins* • Dectin-1 • Mincle • MDL-1 • Mcl • CLEC-2 *NOD-like receptors* • NOD2 • NLRP3 *RIG-like receptors* • RIG-I • MDA5
